# Bioactivity-Guided Fraction from Viscera of Abalone, *Haliotis discus hannai* Suppresses Cellular Basophils Activation and Anaphylaxis in Mice

**DOI:** 10.4014/jmb.2310.10015

**Published:** 2023-11-20

**Authors:** Kap Seong Choi, Tai-Sun Shin, Ginnae Ahn, Shin Hye Kim, Jiyeon Chun, Mina Lee, Dae Heon Kim, Han-Gil Choi, Kyung-Dong Lee, Sun-Yup Shim

**Affiliations:** 1 Department of Food Science and Technology, Sunchon National University, Suncheon 57922, Republic of Korea; 2 Division of Food and Nutrition, Chonnam National University, Gwangju 61186, Republic of Korea; 3 Department of Marine Bio-Food Sciences, Chonnam National University, Yeosu 59626, Republic of Korea; 4 College of Pharmacy and Research Institute of Life and Pharmaceutical Sciences, Sunchon National University, Suncheon 57922, Republic of Korea; 5 Department of Biomedical Science, Sunchon National University, Suncheon 57922, Republic of Korea; 6 Faculty of Biological Science and Institute for Environmental Science, Wonkwang University, Iksan 54538, Republic of Korea; 7 Department of Companion animal industry, College of Health & Welfare, Dongshin University. Naju 58245, Republic of Korea

**Keywords:** Abalone male viscera, basophils, anaphylaxis, NF-κB, cytokine

## Abstract

Basophils and mast cells are specialized effector cells in allergic reactions. *Haliotis discus hannai* (abalone), is valuable seafood. Abalone male viscera, which has a brownish color and has not been previously reported to show anti-allergic activities, was extracted with acetone. Six different acetone/hexane fractions (0, 10, 20, 30, 40, and 100%) were obtained using a silica column via β-hexosaminidase release inhibitory activity-guided selection in phorbol myristate acetate and a calcium ionophore, A23187 (PMACI)-induced human basophils, KU812F cells. The 40% acetone/hexane fraction (A40) exhibited the strongest inhibition of PMACI-induced-β-hexosaminidase release. This fraction dose-dependently inhibited reactive oxygen species (ROS) production and calcium mobilization without cytotoxicity. Western blot analysis revealed that A40 down-regulated PMACI-induced MAPK (ERK 1/2, p-38, and JNK) phosphorylation, and the NF-κB translocation from the cytosol to membrane. Moreover, A40 inhibited PMACI-induced interleukin (IL)-1β, IL-6, and IL-8 production. Anti-allergic activities of A40 were confirmed based on inhibitory effects on IL-4 and tumor necrosis factor alpha (TNF-α) production in compound (com) 48/80-induced rat basophilic leukemia (RBL)-2H3 cells. A40 inhibited β-hexosaminidase release and cytokine production such as IL-4 and TNF-α produced by com 48/80-stimulated RBL-2H3 cells. Furthermore, it’s fraction attenuated the IgE/DNP-induced passive cutaneous anaphylaxis (PCA) reaction in the ears of BALB/c mice. Our results suggest that abalone contains the active fraction, A40 is a potent therapeutic and functional material to treat allergic diseases.

## Introduction

Allergic diseases have become an important public health problem worldwide as individuals develop allergic disorders manifesting as allergic rhinitis or food allergy. These conditions are related to the development of multiple chronic degenerative diseases involving atopic dermatitis, chronic-obstructive pulmonary disease, and chronic asthma, however, an effective treatment for these diseases has not been developed yet [1-3].

Lymphocytes (T and B cells) and granulocytes (basophils, mast cells and eosinophils) play an important role in allergic reaction. Basophils function as specialized effector cells in biological reaction involving immediate type hypersensitivity reactions, host defense system against various pathogens and non-specific immune response [4, 5]. These cells possess cytoplasmic granules containing various inflammatory mediators, including, chemotactic factors, histamine, β-hexosaminidase, prostaglandins, and leukotrienes [6-8]. Basophils interact with various antigens and stimuli involving phorbol myristate acetate (PMA), calcium ionophore (CI), A23187, compound (com) 48/80, and FcεRI α chain-specific antibody in allergic reactions. Activated basophils induce early phase reactive oxygen species (ROS) production, calcium mobilization and early signaling events involving activation of mitogen-activated protein kinases (MAPK). MAPK, extracellular signal-regulated kinase (ERK), p38, and c-jun-N-terminal kinase (JNK) serve as critical activators of downstream transcriptional factors such as NF-κB, which significantly induces inflammatory cytokines and chemokine transcription in allergic reaction. This signaling cascade is essential for degranulation of inflammatory mediators involving histamine, and β-hexosaminidase in activated basophils and mast cells [9-15].

Abalone (*Haliotis discus hannai*) is a single-shelled marine gastropod, which is highly commercialized and represents the most popular seafood resource with sensory (taste and distinctive texture), nutritive, and pharmaceutical value. It has a steady growth tendency owing to global production and consumption. Abalone viscera, which is the by-product that is often discarded as industrial waste, may account for approximately 15 ~ 25% of the total body weight of raw abalone and is used to prepare Jeonbokjuk in South Korea. It contains proteins, polysaccharides, and fatty acids and exhibits tumor, inflammation, oxidation protective effects [16-19]. We previously reported that the active fraction derived from abalone male viscera (AMV) with anti-oxidant activities shows inhibitory effect via inhibition of lipopolysaccharide (LPS)-induced macrophage activation [20]. However, the anti-allergic effects and it’s mechanism underlying the bioactivity of abalone viscera have yet to be examined. The objective of this study was to examine the anti-allergic effects of AMV based on basophil activation using KU812F stimulated by PMACI and RBL-2H3 stimulated by com 48/80 on cellular level, and anaphylaxis in mice.

## Materials and Methods

### Sample Preparation

Raw abalone viscera were purchased from Wando Abalone Co., Ltd., Jeollanam-do, Korea and segregated based on male and female sex. The samples were subject to extraction with acetone and fractionated using six solvent concentrations by varying the acetone/hexane ratio as described previously [20].

### Cell Culture

Human and rat derived basophils, KU812F and RBL-2H3 cells were cultured in RPMI-1640 and DMEM media containing 10% heat-inactivated FBS (Gibco BRL, USA), and antibiotics and antimycotics (Gibco BRL) under 37°C in a humidified atmosphere with 5% CO_2_, respectively. Additionally, the cells were pretreated with 10, 25, 50, and 100 μg/ml A40 for indicated time and stimulated with 40 nM PMA (Sigma-Aldrich, USA) and 1 μM A23187 (Sigma-Aldrich) (PMACI) for KU812F cells and 10 μg/ml com 48/80 (Sigma-Aldrich) for RBL-2H3 cells under serum-free media.

### Cytotoxicity Assay

Cytotoxicity of A40 was evaluated using the MTS assay following the method of the previous study [21]. Briefly, KU812F and RBL-2H3 cells were treated with A40 for 1 h, and stimulated with PMACI and com 48/80 for 24 h in serum-free condition, respectively. And then, MTS solution was replaced to each well and reacted at 37°C for 1 h, and was measured at 490 nm using a microplate reader (Epoch-BioTek Instruments, USA).

### Degranulation Assay

The amount of β-hexosaminidase released in the medium was measured to investigate the inhibitory effects of A40 on degranulation of effector cells. The pretreated KU812F and RBL-2H3 cells with A40 for 24 h were conducted the stimulation with PMACI and com 48/80 in Sinagarian buffer (5 mM KCl, 119 mM NaCl, 5.6 mM glucose, 1 mM CaCl_2_, 0.4 mM MgCl_2_, 25 mM HEPES (Sigma-Aldrich), and 0.1% BSA(Sigma-Aldrich;, pH 7.2), respectively. The upper layer was reacted with 1 μM p-nitrophenyl-N-acetyl-μ-D-glucosaminide (Sigma-Aldrich) at 37°C for 1 h and the reaction was terminated by addition of an equal volume of 0.1 M sodium carbonate buffer (pH 10.0) and was measured at 405 nm.

### Intracellular ROS Assay

The inhibitory effect of A40 against intracellular ROS production was investigated using DCFH-DA (Sigma-Aldrich). Cells (1 × 10^4^ cells/well) were pretreated with various concentration of A40 for 2 h and were stimulated with PMACI for 2 h. The cells were reacted with 25 μM DCFH-DA in HBSS buffer (Sigma-Aldrich) for 30 min and were measured at Ex 485 and Em 538 nm using a fluorescence microplate reader (Molecular Devices Co., USA).

### [Ca^2+^]*i* Level Assay

The inhibitory effect of A40 against [Ca^2+^]*i* level was assessed using Fura 2-AM (Abcam, Danvers, MA, USA). KU812F cells were treated with A40 for 24 h and then loaded with 2 μM Fura 2-AM in HBSS (Sigma-Aldrich) for 30 min. Pretreated and Fura 2-AM loaded KU812F cells were then stimulated with PMACI, and was measured at Ex 405 nm and Em 519 nm.

### Western Blot Analysis

The process of preparing whole cell lysates, cytosolic and nuclear proteins conducting western blotting was performed in accordance with previous study [21]. We used primary antibodies against key targets, involving ERK1/2, p-ERK 1/2, p38, p-p38, JNK, p-JNK, NF-κB and β-actin, and specific HRP-conjugated secondary antibody. Quantitative analysis of each protein band and subsequent data presentation adhered to methodologies outlined in previous study [21].

### Enzyme-Linked Immunosorbent Assay (ELISA)

The production of cytokines, IL-1β, IL-6, and IL-8 in KU812F cells and TNF-α in RBL-2H3 cells was measured using ELISA kits from Invitrogen (Thermo Fisher Scientific, USA). IL-4 production in RBL-2H3 cells was measured using an ELISA kit from R&D systems (USA) according to the manufacturer’s instructions.

### PCA Test

BALB/c male mice (8-week-old), reared under specific pathogen-free conditions, were obtained from Orient Bio (Korea) and all experimental procedures were approved by the Institutional Animal Care and Use Committee of Chonnam National University (No. CNU IACUC-YS-2021-5). The in vivo effects of A40 were investigated in a PCA mouse model. First, 500 ng of anti-DNP-IgE (Sigma-Aldrich) was intradermally injected into the dorsal skin of both ears. Then, 20 μl of A40 (50 and 100 μg/mouse) was tropical applicated to the mice for 2 h before anaphylaxis induction. The mice were then intravenously treated with 30 μl of 10 μg DNP-BSA saline solution containing 4% Evans blue dye (Sigma-Aldrich) [22]. After 30 min, the mice were euthanized via anesthesia with isoflurane followed by cervical dislocation. The skin tissues were collected from the dorsal ear, soaked in 1 ml formamide (Sigma-Aldrich) overnight at 64°C, and measured at 620 nm.

### Statistical Analysis

Statistiscal comparison of the data was achieved using ANOVA and Duncan's multiple range test using the IBM SPSS Statistics V20. The values are defined as the mean ± standard error (SE), and statistical significance was defined as *p* < 0.05.

## Results and Discussion

### Preparation of Active Fraction from AMV via Bioactivity-Guided Selection

Effector cells such as basophils and mast cells function in allergic reactions, and they possess cytoplasmic granules containing various inflammatory mediators, which are activated by stimuli such as A23187, com 48/80, and anti-FcεRI antibody. The activated effector cells induce the degranulation and subsequent release of inflammatory mediators such as histamine, β-hexosaminidase, leukotrienes, and prostaglandins. Among the substances, β-hexosaminidase is a strong degranulation marker in allergic reactions [4, 7, 8]. In this study, we utilized basophils, KU812F cells isolated from chronic myeloid leukemia patients and stimulator, PMACI for induce the allergic disorders.

Abalone is a food containing several nutrients such as proteins, polysaccharides, and fatty acids, and its components show bioactivities involving inflammation, oxidation, and tumor protective activities; therefore it is widely consumed in Asian countries [16-21]. The present study focused on the viscera, which contains various pigments between male (brown) and female (green) viscera as by-product of abalone.

**Fig. 1:**
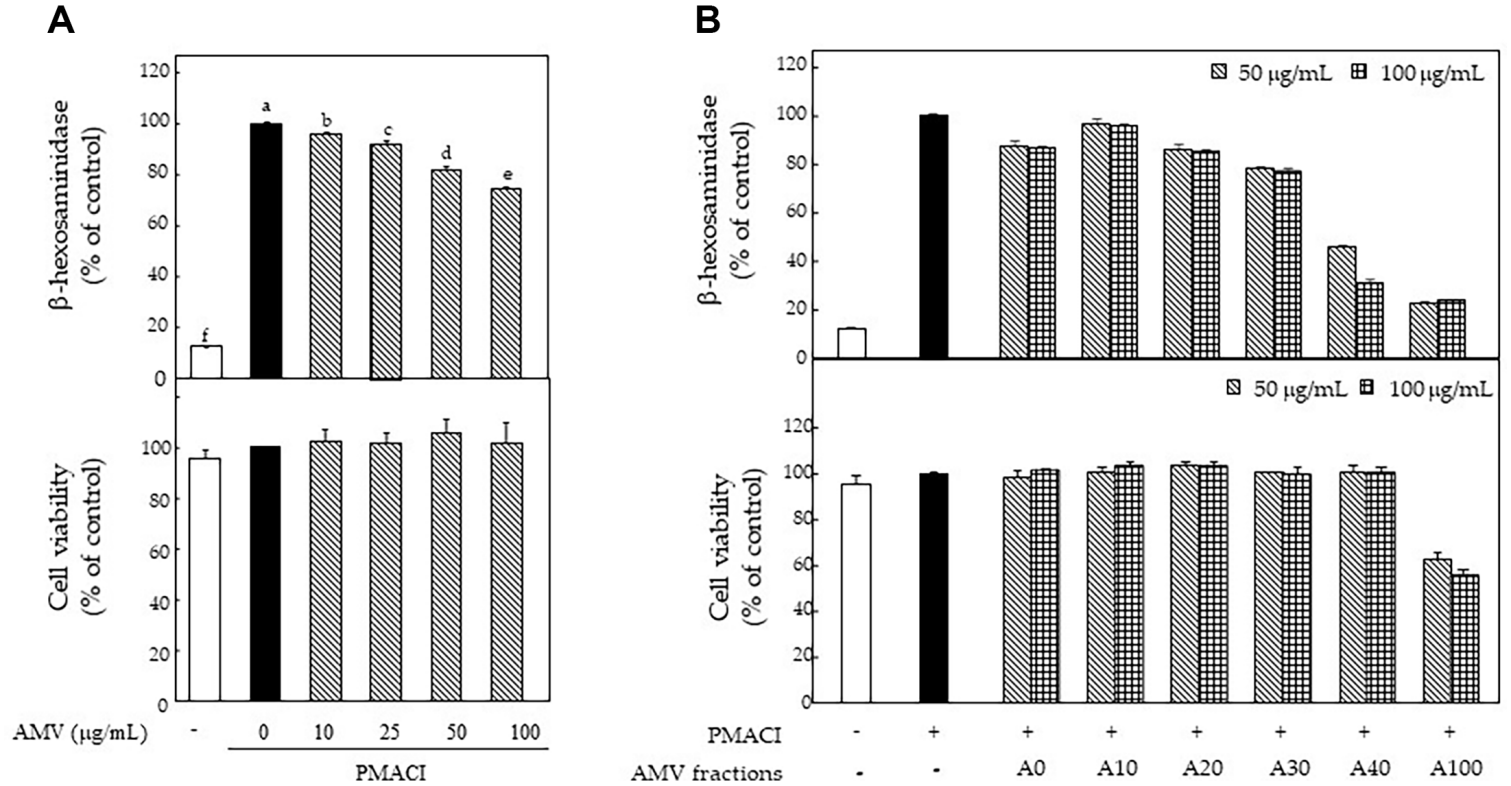
Anti-allergic effects of extract and fractions prepared from AMV. (**A**) β-hexosaminidase level and cell viability of acetone extract from AMV in 40 nM PMA and 1 μM A23187- stimulated KU812F cells, (**B**) β-hexosaminidase level and cell viability of fractions from AMV in 40 nM PMA and 1 μM A23187-stimulated KU812F cells. Values are shown as the mean Data are expressed as the means ± SE of triplicate experiments. The small letters indicate significant differences (*p* < 0.05).

To obtain the active fraction with degranulation inhibitory effects from abalone, the abalone viscera were separated according to the sexes and extracted using acetone, after which the β-hexosaminidase release inhibitory effects were observed in human basophilic KU812F cells using PMACI stimulation. The brownish AMV extract showed more potent inhibitory effects than those of the greenish abalone female viscera (data not shown) and dose-dependently inhibited the PMACI-induced β-hexosaminidase release (Fig. 1A). Subsequently, the AMV extract was fractionated using acetone/hexane ratios using a silica column as previously reported. Among the fractions obtained , A40 was an active fraction with the most potent inhibitory effects of β-hexosaminidase release without cytotoxicity in PMACI-stimulated KU812F cells (Fig. 1B). Therefore, A40 was selected and its anti-allergic activities were assessed based on down-regulation of basophil activation on cellular level and anaphylaxis in mice.

### Effects of A40 on Intracellular ROS Production and Calcium Mobilization in PMACI-Stimulated KU812F Cells

**Fig. 2:**
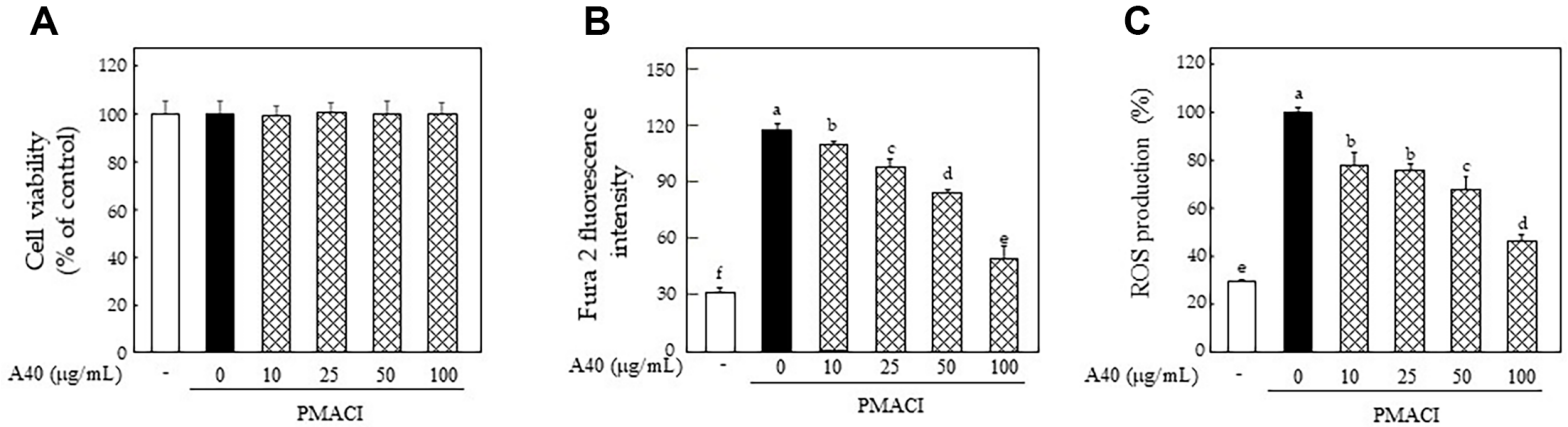
Effects of A40 on [Ca^2+^]*i* level and ROS production in PMACI-stimulated KU812F cells. Cytotoxicity of A40 (**A**), [Ca^2+^]*i* level (**B**), intracellular ROS production in 40 nM PMA and 1 μM A23187-stimulated KU812F cells. Values are shown as the mean Data are expressed as the means ± SE of triplicate experiments. The small letters indicate significant differences (*p* < 0.05).

KU812F cells was examined using an MTS assay to determine the non-toxic level of A40. The fraction did not show any cytotoxicity in PMACI-stimulated KU812F cells at levels up to 100 μg/ml in comparison with non-stimulated cells after 24 h of treatment (Fig. 2A).

Basophil activation induces various cellular responses, including calcium influx and intracellular ROS production in response to various stimuli including FcεRI crosslinking, com 48/80, and PMACI.

Free calcium ions play a pivotal role in the initiation and modulation of secretory action in allergic reactions, thereby activating their Ca^2+^ channel function such that Ca^2+^ flows from the endoplasmic reticulum into the cytosol [23].

ROS, which is important in host defense of inflammatory and immune responses and is related to the progression of various diseases, is generated by an breakage of balance between antioxidant and pro-oxidant homeostasis and depends on generation and removal by the antioxidant system [9, 11, 24]. We investigated the inhibitory effects of A40 on calcium influx and intracellular ROS production in PMACI-induced KU812F cells using the specific probes, Fura 2-AM and DCFH-DA, respectively. [Ca^2+^]*i* and ROS levels increased with PMACI stimulation, whereas A40 treatment dose-dependently reduced [Ca^2+^]*i* (Fig. 2B) and ROS levels (Fig. 2C) without showing any cytotoxicity.

### Effects of A40 on Protein Expression of MAPK and NF-κB Signaling in PMACI-Stimulated KU812F Cells

**Fig. 3:**
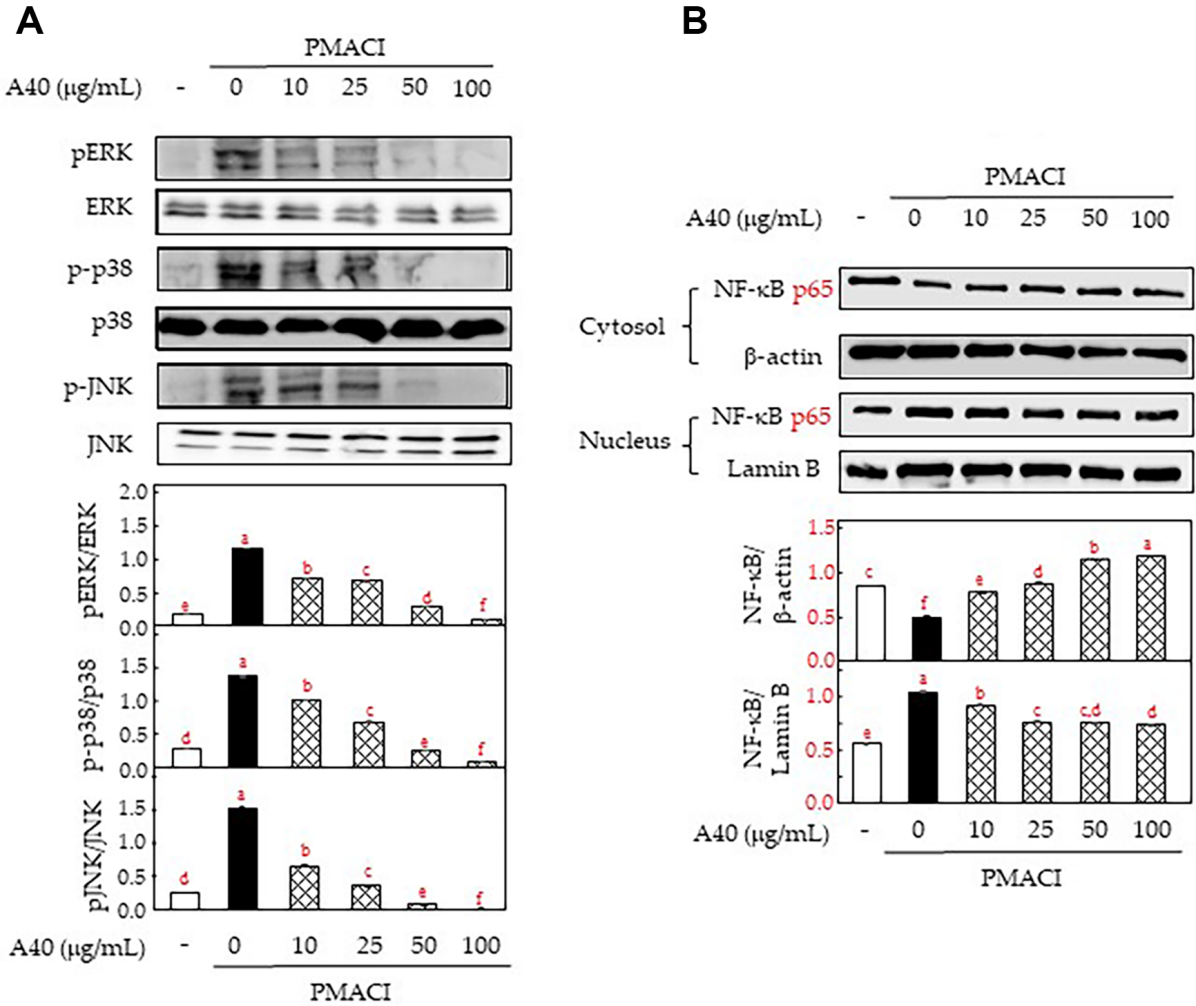
Effects of A40 on MAPK and NF-κB activation in PMACI-stimulated KU812F cells. MAPKs phosphorylation (**A**), p65 translocation (**B**) in 40 nM PMA and 1 μM A23187-stimulated KU812F cells for 1h. Values are shown as the mean Data are expressed as the means ± SE of triplicate experiments. The small letters indicate significant differences (*p* < 0.05).

We investigated the effects of A40 on the signaling of MAPK and NF-κB in PMACI-stimulated KU812F cells via western blot analysis. PMACI stimulation induced the increment of the expression levels of phosphorylated ERK, p38, and JNK in KU812F cells, whereas they were markedly dose-dependently reduced by the A40 pretreatment (Fig. 3A). Further, the PMACI stimulation induced the translocation of NF-κB from the cytosol to the nucleus. we determined that A40 pretreatment effectively modulated NF-κB translocation in PMACI-stimulated KU812F cells (Fig. 3B). These results suggest that A40 effectively regulated the MAPK and NF-κB signaling in activation of KU812F cells by PMACI.

Activation of effector cells such as basophils and mast cells is related to the up-regulation of various signaling transcriptional-factors in allergic reactions that include protein tyrosine kinases (Syk, Lyn, and Fyn), protein kinase C, and phosphoinositide 3-kinase [25-28]. To better understand the inhibitory mechanism of A40 in the activation of basophils and mast cells, further studies should investigate the regulation of the signaling transcriptional factors.

### Effects of A40 on Cytokine Production

**Fig. 4:**
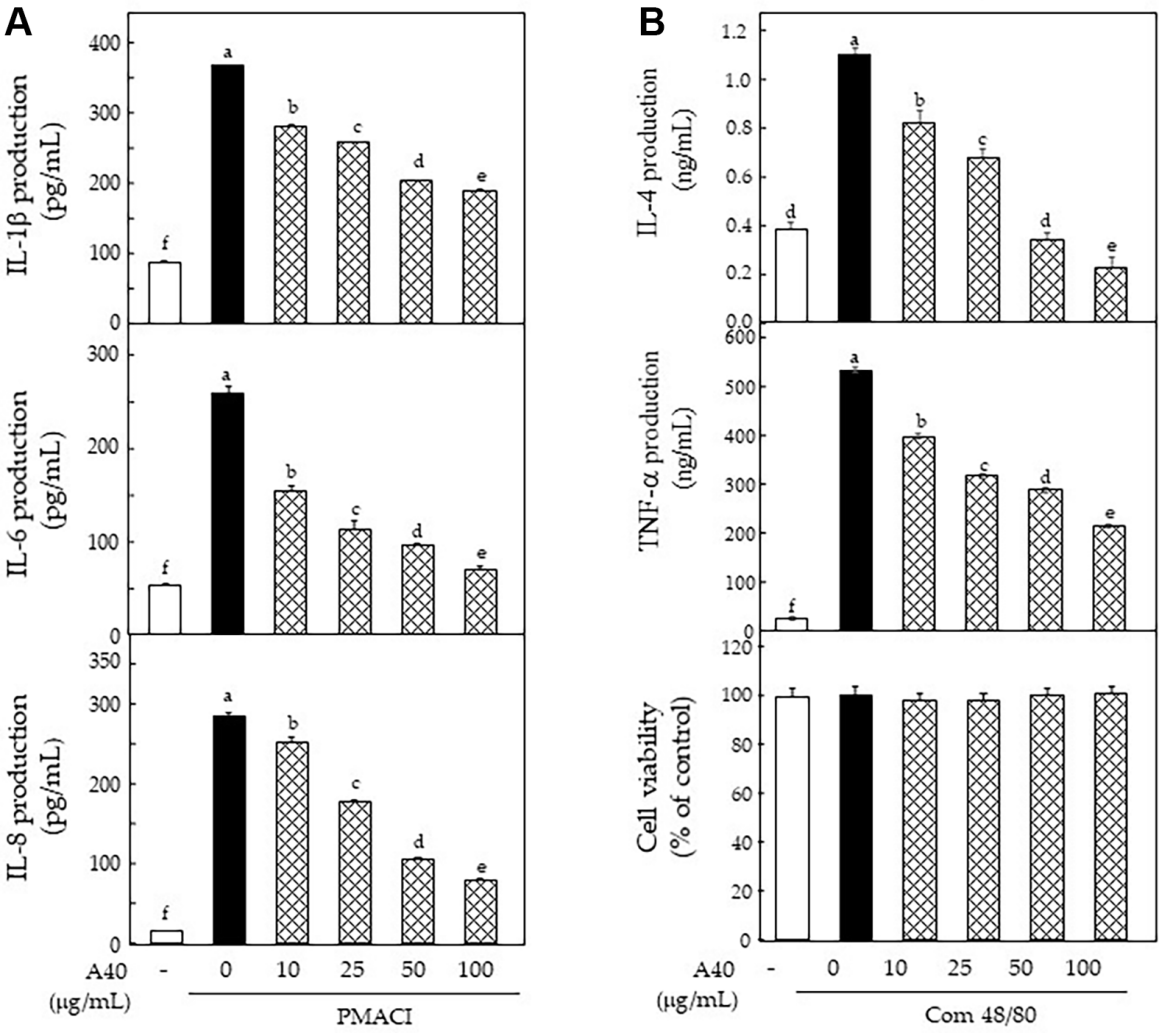
Effects of A40 on cytokines production in PMACI-stimulated KU812F and com 48/80-stimulated RBL-2H3 cells. Production of cytokine 40 nM PMA and 1 μM A23187-stimulated KU812F (**A**) and 10 mg/ml com 48/80-stimulated RBL-2H3 (**B**) for 24 h. Values are shown as the mean Data are expressed as the means ± SE of triplicate experiments The small letters indicate significant differences (*p* < 0.05).

MAPK phosphorylation and NF-κB translocation are essential for upregulating cytokines in basophil activation [14, 15, 27, 28]. We examined the inhibitory effects on cytokine production in A40-treated and PMACI-stimulated KU812F cells using ELISA. PMACI stimulation induced a remarkable increment in production of IL-1β, IL-6, and IL-8 (Fig. 4A). To confirm the inhibitory effects of A40 on basophil activation, the media of RBL-2H3 cells, pretreated with A40 and stimulated with com 48/80, were used to examine cytokine production. A non-toxic concentration of A40 in com 48/80-stimulated RBL-2H3 cells, which was measured via MTS assays, was found to be 100 μg/ml when comparing cells with non-stimulated cells after 24 h of treatment (Fig. 4B). Com 48/80 stimulation markedly increased the production of IL-4 and TNF-α, compared to that in non-stimulated cells. Notably, these proteins were effectively reduced via treatment of A40 (Fig. 4C).

IL-1β, IL-6, and TNF-α are represent characteristic cytokines in allergic inflammatory disorders and proinflammatory cytokines generated by activated effector cells with PMACI, com48/80, LPS, and anti-FcεRI antibody [28-30]. IL-4 firstly produced by activation of basophils and mast cells in allergic reactions. Our ELISA results showed that A40 inhibited the production of these cytokines.

Cytokines, growth factors, and chemokines, which are secreted by activated immune cells, mediated various immune responses. More studies are needed to evaluate the role of these factors upon activating effector cells involving basophils and mast cells.

### Effects of A40 on Degranulation

Activated basophils and mast cells can secrete inflammatory mediators including histamine, β-hexosaminidase, serotonin, prostaglandins, and leukotrienes, which are characteristic for allergic reactions [31]. The levels of these substances released in the media can serve as degranulation markers of basophils and mast cells. Among the inflammatory mediators such as lysosomal enzymes, β-hexosaminidase are potent degranulation marker of basophil activation in allergic diseases. We determined that A40 dose-dependently inhibited PMACI-induced β-hexosaminidase in KU812F cells.

**Fig. 5:**
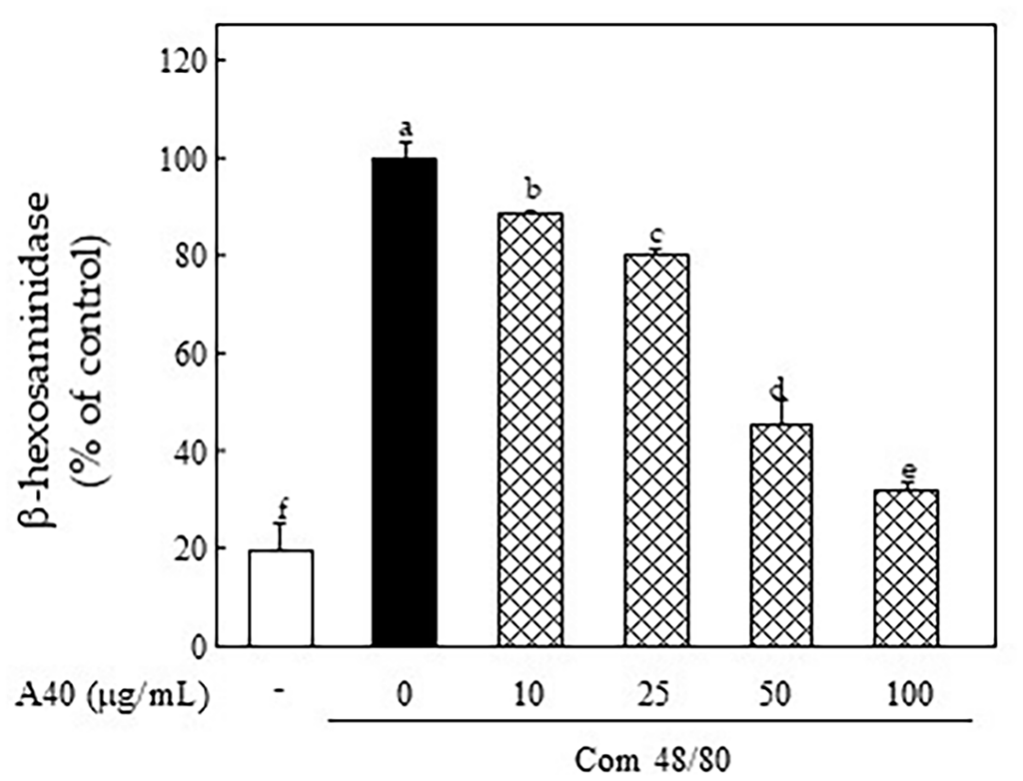
Effect of A40 on basophil degranulation in com 48/80-stimulated RBL-2H3 cells. β-hexosaminidase release in 10 μg/ml com 48/80-stimulated RBL-2H3 cells for 1 h. Values are shown as the mean Data are expressed as the means ± SE of triplicate experiments. The small letters indicate significant differences (*p* < 0.05).

To confirm the protective effects of A40 on basophil activation, we investigated the com 48/80-induced β-hexosaminidase release in RBL-2H3 cells. Com 48/80 stimulation induced increment the release of β-hexosaminidase from RBL-2H3 cells, whereas it was dose-dependently reduced via A40 pretreatment (Fig. 5).

### Effects of A40 on DNP/IgE-Induced PCA in Mice

PCA, which results in increase of vascular permeability in the skin and can be examined via intravenous injection of Evans blue, is characterized by an immediate skin reaction caused by a localized IgE-mediated allergic reaction in vivo [22, 32]. Ear model of PCA mouse was used to examine the effects of A40 on IgE-mediated allergic response in an in vivo animal model. IgE stimulation simultaneously accompanied by a strong PCA reaction with the increased Evans blue dye amount, compared to that in non-stimulated mice. Notably, the A40 treatment reduced the amount of the Evans blue extracted from mouse ear skin in an IgE-induced PCA mouse model. Therefore, we show that the anti-allergic activity of A40 in PCA in vivo model.

Our study firstly showed that A40 can inhibit basophil activation on cellular level and anaphylaxis in mice. Further studies on FcεRI-mediated molecular-protective mechanisms of A40 are needed to confirm the function as functional food materials that use abalone viscera, which shows potent oxidation and inflammation protective activities, to protect against allergic disorders.

## Conclusion

Taken together, this study demonstrates that A40 fractionated from AMV shown inhibitory effects of basophil activation on cellular level and PCA reaction in mice. These results firstly showed that A40 could inhibited activation in human basophilic KU812F cells following exposure to PMACI, involving ROS production, calcium mobilization, degranulation, and cytokine production. Furthermore, these protective effects of A40 on KU812F cells activation were related to the down-regulation of transcription factors such as ERK, p38, JNK, and NF-κB. We confirmed that A40 inhibited com 48/80-induced degranulation and cytokine production in RBL-2H3 cells. Moreover, A40 exerted an anti-allergic effect in IgE/DNP-induced PCA mice. Altogether, our results suggest that AMV containing active fraction, A40, is a potential pharmaceutical agent and functional food material.

## Figures and Tables

**Fig. 6:**
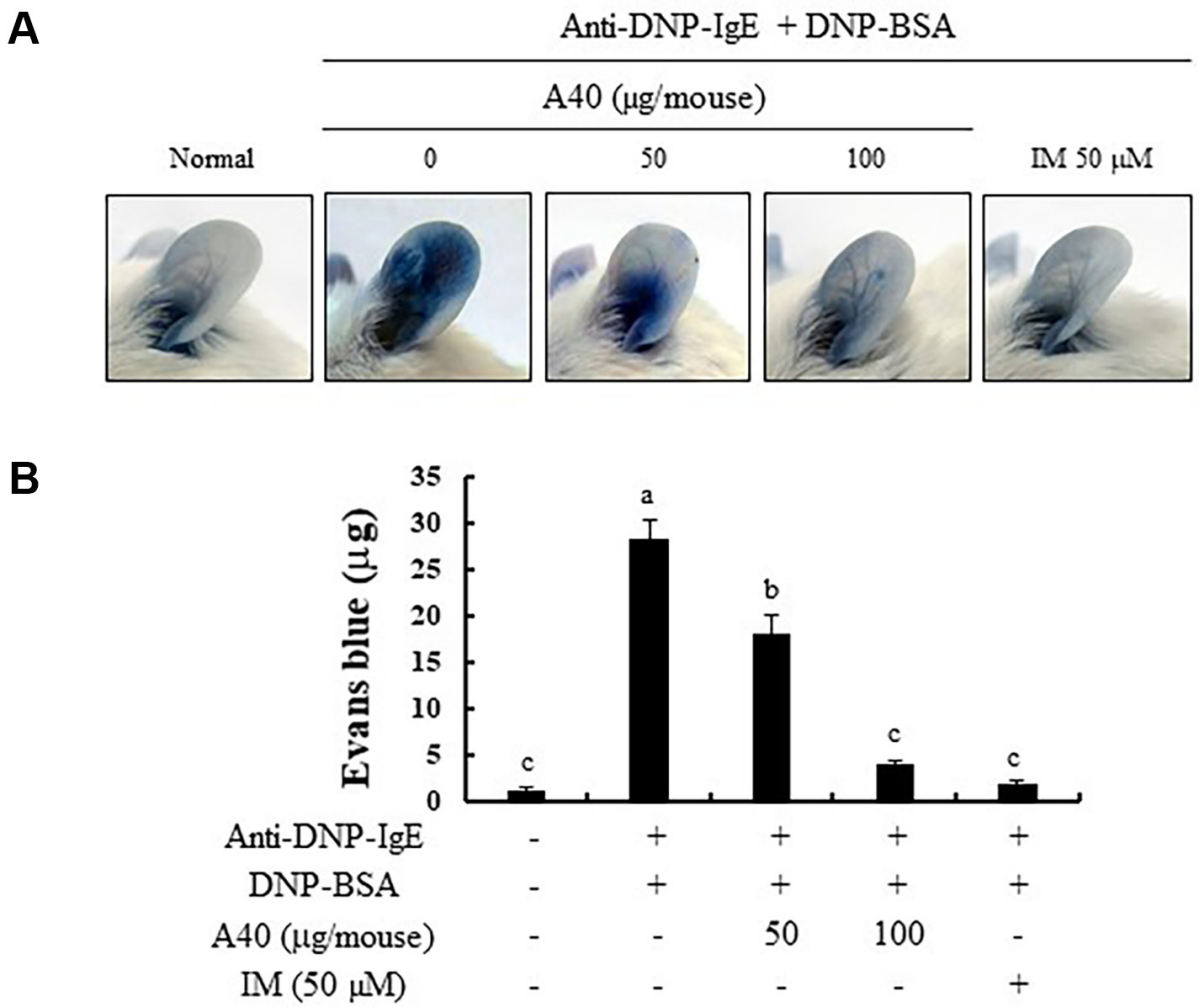
Effect of A40 on the IgE-mediated PCA reaction in mice. (**A**) Representative photographic images of ears, (**B**) Amount of extracted dye. Values are shown as the mean Data are expressed as the means ± SE of triplicate experiments. The small letters indicate significant differences (*p* < 0.05).
